# Towards Aircraft Maintenance Metaverse Using Speech Interactions with Virtual Objects in Mixed Reality

**DOI:** 10.3390/s21062066

**Published:** 2021-03-15

**Authors:** Aziz Siyaev, Geun-Sik Jo

**Affiliations:** 1 Artificial Intelligence Laboratory, Department of Electrical and Computer Engineering, Inha University, Incheon 22212, Korea; azizsiyaev.ai@gmail.com; 2 Augmented Knowledge Corp., Inha Dream Center, 100 Inha-ro, Michuhol-gu, Incheon 22212, Korea

**Keywords:** metaverse, mixed reality (MR), aircraft maintenance education, speech interaction, deep learning, Boeing 737, Industry 4.0, smart maintenance

## Abstract

Metaverses embedded in our lives create virtual experiences inside of the physical world. Moving towards metaverses in aircraft maintenance, mixed reality (MR) creates enormous opportunities for the interaction with virtual airplanes (digital twin) that deliver a near-real experience, keeping physical distancing during pandemics. 3D twins of modern machines exported to MR can be easily manipulated, shared, and updated, which creates colossal benefits for aviation colleges who still exploit retired models for practicing. Therefore, we propose mixed reality education and training of aircraft maintenance for Boeing 737 in smart glasses, enhanced with a deep learning speech interaction module for trainee engineers to control virtual assets and workflow using speech commands, enabling them to operate with both hands. With the use of the convolutional neural network (CNN) architecture for audio features and learning and classification parts for commands and language identification, the speech module handles intermixed requests in English and Korean languages, giving corresponding feedback. Evaluation with test data showed high accuracy of prediction, having on average 95.7% and 99.6% on the F1-Score metric for command and language prediction, respectively. The proposed speech interaction module in the aircraft maintenance metaverse further improved education and training, giving intuitive and efficient control over the operation, enhancing interaction with virtual objects in mixed reality.

## 1. Introduction

Metaverse is coming [1]. The world started to experience an extraordinary mixed reality (MR) digital place inside of the physical world where people seamlessly get together and interact in millions of 3D virtual experiences. What is more, the coronavirus pandemic [2], "with its requirements of physical distancing, has brought people into online digital environments for a growing range of shared human experiences" [1]. Soon being a part of daily life, metaverses will also have a key role in whole industries, such as aviation maintenance. Transforming physical aircraft that are worth hundreds of millions of dollars (i.e., Boeing 737 Max 8 costs $121.6 million [3]) into virtual digital twins will allow airlines and maintenance repair operation (MRO) service providers to save a lot of money and resources and increase the productivity, efficiency, and quality of the maintenance process by enhancing technicians’ workflow with mixed reality collaboration. In addition, with virtual models that can be easily downloaded, maintenance crew members can share the content around the globe, and train fellow engineers in another part of the world in real-time, using augmented virtual aircrafts available to everyone. Airlines or aviation schools that use old physical units for training purposes will soon replace equipment and aircrafts with modern virtual 3D models that are dynamic and interactive with built-in manuals, warnings, and tutorial instructions to follow.

Recently, in the emerging world of mixed reality applications, various industries are already gaining advantages from these technologies [4,5,6,7,8,9]. Specifically, MR has a special role in maintenance since it helps professional field technicians to cope with high-degree worker-centered processes and heavy cognitive load; it helps to visualize information, analyze documents easier, and support the decision-making during operations [5]. Mixed reality applications powered by head-mounted devices such as smart glasses such as Microsoft HoloLens 2 [10] give opportunities to perform work with both hands without holding a mobile device such as a smartphone and tablet. This triggers the importance of voice interaction using speech commands, which gives the ability to control the workflow without having to click buttons or use hand gestures. In addition, speech interaction in smart glasses minimizes the user’s efforts to access certain information, overall, speeding up the maintenance process [11]. Mixed reality experiences in the aircraft maintenance metaverse need speech interaction since engineers’ hands are busy with equipment. Besides, when using speech interaction to enhance maintenance training, it creates a feeling of multi-tasking and supervision, which helps to navigate inside this meta world and "talk to" virtual objects and receive feedback. Thus, speech interaction is an essential component in MR-based maintenance to have a seamless workflow. Having said that, the built-in voice module in modern smart glasses has its limitations.

Voice interaction in smart glasses is not flexible; only static, predefined speech commands can be applied. The user needs to pronounce a command precisely to make a system respond. Thus, before using voice interaction, all supported commands must be learned, which creates an additional cognitive load for users [5]. Next, in the case of maintenance, domain-specific vocabulary is not well recognized; commands that include technical words or phrases that reference manuals or equipment are usually misunderstood and replaced with common words. For example, "IPC View" (Illustrated Parts Catalogue usually referred to as IPC) is recognized as "My PC View". What is more, built-in speech recognition is limited to a specific language. In scenarios where users communicate with intermixed commands, such as Korean aircraft maintenance engineers who use documentation in English and combine languages, existing voice modules do not work. This provides a trigger for alternative solutions such as deep learning [12].

Deep learning shows enormous potential in speech command recognition tasks [12]. We analyzed the literature on speech and sound recognition for mobile devices since, to the extent of our knowledge, there are no similar related works that worked on custom voice interaction models for smart glasses. The most related works, such as [13,14,15,16,17,18,19,20], dealt with single-word simple speech commands, based on the Dataset for Limited-Vocabulary Speech Recognition [21]. The common property they share is the architecture, which is based on convolutional neural networks (CNN) for features extraction and fully connected layers for classification [22]. The authors of [13,14,16,20,23] applied 1D convolutions with raw audio, whereas [13,15,17,18,24,25] used 2D convolutions with special audio features such as mel frequency cepstral coefficients (MFCCs) [26]. According to [13], both approaches are effective, but MFCCs are a great approximation to the human voice that help to discard background noise [26]. Summarizing the limitations of the referenced works, they do not deal with complex commands, datasets they experimented with are monolingual, and their architectures are not suitable for command and language detection at the same time. Therefore, following their best approaches, we built our own speech command recognition deep learning model.

**Figure 1:**
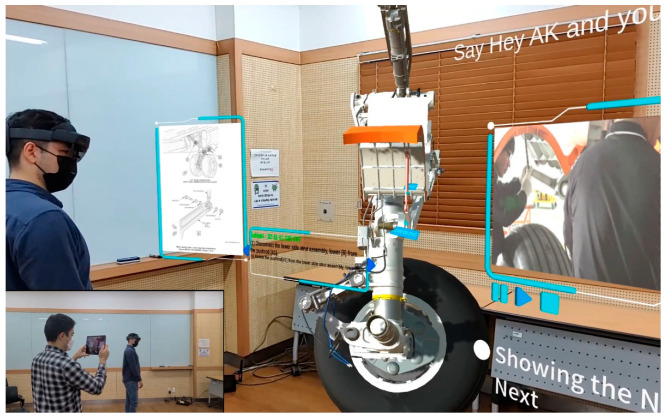
Mixed reality-based maintenance education of landing gear removal of Boeing 737 captured with Spectator View, which enables viewing MR content of HoloLens from secondary devices.

Moving towards the development of aircraft maintenance metaverse, in this work, we built aircraft maintenance education using mixed reality content in Microsoft HoloLens 2 smart glasses enhanced with a proposed speech interaction module that helps to orient inside the virtual Boeing 737 maintenance world (see Figure 1). In Figure 1, the trainee wears a HoloLens 2 and performs the education process of landing gear removal, whereas another student using Spectator View (a special application that allows the visualization of virtual content of reference HoloLens) [27] on a tablet can interact in the virtual space with MR content of his fellow engineer. As can be seen, the physical world combined with virtual 3D assets of aircraft have enormous potential for educational purposes when shared among trainees. Taking into account the fact that aviation colleges currently use retired models of aircrafts that do not have modern technological updates in them, our work makes it possible to reimagine aircraft maintenance education in Industry 4.0 by providing a scalable solution with constantly updated virtual 3D models that can be easily updated, manipulated, downloaded, and shared. What is more, the aircraft 3D visualizations with smart glasses are presented in actual size with all supported equipment, maximizing the effect of the presence, delivering industry-level experience.

**Figure 2:**
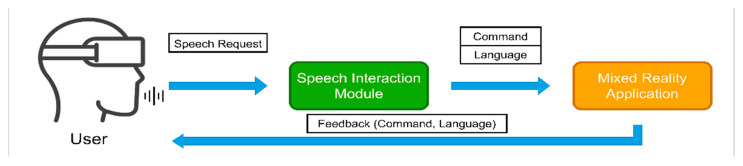
Speech interaction flow with mixed reality application.

In addition, combining cutting-edge technologies such as mixed reality and artificial intelligence in the era of Industry 4.0, we built a speech interaction module powered by a deep learning model that enables trainees "to talk" with mixed reality content and control the workflow of the maintenance process. Speech interaction creates a tremendous possibility to imitate an expert in the field of aircraft maintenance that guides trainees during the education process and facilitates tutoring by providing required materials such as technical manuals, instructions, and reference videos that support maintenance education. We addressed the above-mentioned limitations of voice interaction in smart glasses and created a custom voice interaction module, which is flexible enough to recognize the wide variation of speech commands for operation and supports intermixed requests in English and Korean languages, recognizing the corresponding language of command (see Figure 2). First, we collected a dataset of user commands in English and Korean languages. Next, we created a deep learning-based model that predicts the requested command and the language in which the command was spoken. The network has customized architecture that uses CNN for audio features learning, and two output classification parts for commands and language identification, respectively. The module records speech commands by the user and extracts MFCC features for inference. Based on the speech interaction module result (see Figure 2), the MR application performs a particular action, and voice feedback is given back to a user in the predicted language (i.e., if a person makes a speech request in Korean, the response from the application will be in Korean, accordingly). Finally, we embedded the model into the HoloLens smart glasses and performed an evaluation process.

To evaluate the performance of the speech interaction module, we created several sets of test data, including intermixed commands, and separate English and Korean test sets. To carry out the quantitative analysis, classification metrics such as accuracy, precision, recall, and F1-Score, confusion matrix applied, and experimental results demonstrated that the model can accurately identify the commands’ type along with language, showing an average F1-Score 95.7 and 99.6% for command and language recognition, accordingly. The model has only 181,000 weight parameters, taking up a small memory size of 716kB with real-time performance on smart glasses. Overall, the proposed speech interaction module is enhancing aircraft maintenance with smart glasses by giving trainee-engineers a flexible, intuitive, and productive way to easily navigate inside the maintenance routine. Furthermore, the module works properly even when a user wears a face mask (see Figure 1), coping with various background noises and speech uncertainties due to a covered mouth, which is practically demanding in times of social distancing.

In the following sections, we described the importance and breakthrough of mixed reality in the field of maintenance and education. Next, we discussed deep learning techniques for developing a custom speech interaction module. In Section 3, we presented the proposed mixed reality environment for aircraft maintenance education and training. Section 4 and Section 5 presented step-by-step explanations on the dataset collection, audio features extraction, and the network architecture of the proposed module. Lastly, we demonstrated evaluation techniques and experimental results to make a conclusion.

## 2. Background

### 2.1. Mixed Reality in Maintenance and Education

Mixed reality is becoming a new player in the maintenance industry, creating great potential for industrial applications [4,5,6,7,8,9]. Engineers that use MR content guidance in various head-mounted devices such as smart glasses can seamlessly perform work with both hands and enhance their workflow. Taking the case of maintenance in the aviation industry, MR is essential, due to a high degree of worker-centered processes. Here, various inspection and maintenance processes are characterized by a high percentage of manual work steps, a great variety of handled components, and a considerable effort for documentation. Therefore, the cognitive support systems based on MR can deliver great value to the workers [5]. For example, the use of MR for visualization will allow the analysis, interaction, and exploration of information easier, hence facilitating operators’ decision-making during maintenance tasks [7].

Mixed reality provides mobility. In the specific context of an application scenario, maintenance personnel should be highly mobile; while performing operational and maintenance tasks at different maintenance facilities or directly on the field, MR creates the possibility to work independent of location, while still following the instructions to perform the repairment process [4]. Digitalized aircraft can be easily downloaded to a working device and can easily be moved in the space at the touch of a finger. Usually, at the maintenance site, in order to manipulate the physical part of a massive machine, special equipment is required, however, MR opens up enormous advantages for mobile exploration.

Mixed reality improves collaboration. Considering that maintenance is a synergetic process, where more than two participants are involved, according to [28], the presence of virtual objects in the physical world improves spatial cues in collaborative mixed reality environments (MREs). Engineers who work in MREs experience a positive influence on collaborators’ communication behavior for object identification and object positioning tasks. The authors of [28] suggest that adding virtual objects to MREs “reduces user task load and improves groups’ communication and user experience”. As a result, while performing certain work, group members used more informative verbal references rather than deictic gestures, decreasing the subjective workload when virtual objects were present.

MR can facilitate training and access to large amounts of documentation. The authors of [8] present work in training and assisting in maintaining equipment in complex industrial environments. As the complexity of the assembly or maintenance task increases, training becomes a significant factor with respect to both time and money. Such a technology as MR can make an enormous addition to tutoring future technicians by giving them the ability to interact with virtual objects but receive nearly the same experience as if they worked with real ones. Taking the example of the most exploitable aircraft with over 10,000 active units, the Boeing 737 [29]: the flight cost of Boeing 737-800 is about $2180 per hour [30], which means whenever an aircraft stays on land for MRO, it is extremely costly for the owner/company. In this sense, MR is an effective way to educate employees and at the same time save money, since a physical plane will not be required.

To gain the best experience from mixed reality, smart glasses, which overlay content in front of the eyes at a wide-angle and give the ability to work with both hands and no longer hold a device, should be used. Moreover, engineers that wear smart glasses should receive high-level human–computer interaction that speeds up the maintenance process, not slows it down. Therefore, interactions in smart glasses such as gestures, eye-tracking, and voice should be developed in a user-friendly way. What is more, depending on the application, one interaction type might play a key role. Taking into consideration the nature of the maintenance work, where technicians use both hands to complete tasks, voice interaction using various speech commands is crucial to control the MR since you do not have to click buttons or use hand gestures, thus enabling the ability to have a seamless interaction with the system.

### 2.2. Smart Glasses Voice Interaction 

Voice input in smart glasses brings colossal benefits to its users. The authors of [11] show that voice interaction with a system can significantly reduce a person’s cognitive load since there is no need to remember the steps of particular action invocations; moreover, it minimizes the user’s efforts by making tasks effortless. Taking an example of the voice input of the current most-advanced head-mounted device for mixed reality, Microsoft HoloLens 2 [10], voice communication is great for interacting with traversals to cut multiple actions, making users experience powerful emotional effects whilst saving a lot of time. What is more, it gives a sense of multi-tasking whenever users’ arms are busy [11]. Considering these opportunities that smart glasses have, there are also technical limitations. 

First, system-built voice interaction is based on static pre-defined commands. When certain voice commands are set to invoke a particular action, a person wearing the smart glasses must speak out the command in exactly the way it was programmed. For example, if "next instruction" is defined in the system, then "alright, show me the next instruction" is not going to work. Users must either remember all the commands exactly how they are set, which is a contradiction to the user-friendliness paradigm, or developers need to handle this with multiple variations of the same voice command in the system, which is inconvenient to maintain when there are many control functions.

Second, built-in speech recognition is not suitable for a domain-specific language. Taking the example of maintenance in the aviation industry, there exist special manuals such as the IPC (Illustrated Parts Catalogue), and when the dictation speech input option is invoked in the HoloLens 2 smart glasses, the speech command "show me IPC view" is recognized as "show me my PC view". Thus, in the cases where certain professional words are called, there is no guarantee that the built-in speech recognition feature performs well.

Third, voice interaction is not optimized for intermixed commands, where a sentence consists of words that belong to two or more languages. Coming to the industry again, companies and employees around the world always exchange their technologies; therefore, it is a usual case when professional vocabulary in a certain community consists of words from various languages. In the case of aviation maintenance, all manuals are in English; therefore, using English domain-specific words during the work process by, for example, Korean engineers is unavoidable.

Even though advancements in smart glasses, particularly voice interaction, bring value to users and cope with basic needs and functionality, it might not be enough to deliver to an industry a scalable, dynamic, and flexible approach that can recognize speech commands with wide variations that includes words from the domain-specific vocabulary. Therefore, in this work, we applied an alternative approach to handle these limitations and fulfill our requirements with deep learning.

### 2.3. Deep Learning for Speech Interaction

Deep learning is the pick. According to [12], deep learning-based approaches show better performance over traditional machine learning methods for many different applications. They demonstrate massive potential in various tasks related to audio input, such as speech recognition, sound, and speech classification [13]. In addition, interesting development have occurred in speech enhancement systems based on visual speech recognition when a system needs audio and visual information at the same time for inferencing speech [31], which is beneficial for people with disabilities in hearing. However, according to the requirements of our speech module, we want to classify a single audio speech request into one of the command types and recognize the language; hence, it is not a speech enhancement task nor speech recognition, where you must build the sentence from a speech and have a high level of word or character accuracy. Therefore, sound and speech classification works are further analyzed.

The recent works in speech and sound classification mostly dealt with simple single-word speech commands (A Dataset for Limited-Vocabulary Speech Recognition [21]) or urban sounds (A Dataset and Taxonomy for Urban Sound Research [32]). The fundament of their networks’ architecture is the convolutional neural network (CNN); however, the dimensions of the convolutional block and type of audio feature are divided into two approaches. First, [13,14,16,20,23] used raw audio directly, without extracting features. Since audio is a one-dimensional signal, their CNN architecture is also based on one-dimensional filters. Second, [13,15,17,18,24,25,31] applied various audio features such as mel frequency cepstral coefficients (MFCCs) [26] or Spectrograms with 2D convolutions. Since these features have a two-dimensional representation, CNN filters have the corresponding shape. Even though both approaches perform nearly the same, the analyses in [13,26,33] show evidence that MFCCs are great at the approximation to the human voice, which helps to identify linguistic content and discard background noise or certain emotions present in the audio. Therefore, in our work, we decided to follow the second approach—using 2D CNN-based architecture with MFCC as audio features.

The referenced works in speech command recognition demonstrated the ability to easily handle simple single-word classification; however, the following limitations are present:

1. They do not deal with complex commands classification, where multiple variations of request belong to one class;
2. The datasets they experiment with and trained networks are monolingual. In our work, we work with English, Korean, and intermixed commands at the same time;
3. Their networks’ output layers only give the command class; however, we need to identify the command type and language of command as well.

Therefore, we created our own deep learning network architecture and trained it on a custom collected dataset of spoken commands following their effective practices.

## 3. Mixed Reality Maintenance Education for Boeing 737

Moving towards the development of the aircraft maintenance metaverse, we proposed the mixed reality-based application in the Microsoft HoloLens 2 smart glasses for maintenance education of the aircraft Boeing 737. Creating a virtual aviation space, MR guides a trainee wearing the smart glasses and overlays aircraft-specific mixed reality content and other supportive materials in front of the user’s line of sight, combining assets of both physical and virtual worlds. A user is provided step-by-step instructions to complete a certain task, such as landing gear removal, and all the required documents and manuals, tutorial videos, MR-based illustrations. Since trainee mechanics perform MRO and training procedures, they use both hands, receiving instructions and illustration in the metaverse while work is performed on a physical site. Therefore, the main functions to support operations and allow seamless workflow need to be invoked using speech commands without clicking buttons or using hand gestures, creating a multitasking experience.

### 3.1. Application Overview

**Figure 3:**
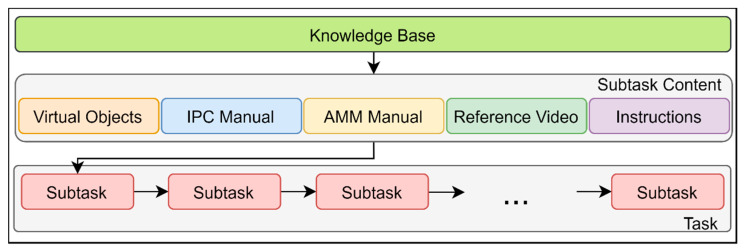
Action & Content Flow of MR.

Aircraft maintenance MRO is performed in stages. There is one global task, such as landing gear removal, which includes subtasks that need to be completed. Each subtask has its own materials to support engineers, and instructions to follow. Figure 3 describes the action and content flow of the maintenance process implemented in our work. According to a subtask that a user works on, subtask content is delivered to the smart glasses. subtask content includes the following (see Figure 3):

- *Virtual objects.* Mixed reality 3D models of aircraft and their specific components. Virtual models contain animations that guide a trainee by showing the proper way of action on a specific part. In addition, certain parts of the virtual model are highlighted since they are referred to in the manuals;
- *IPC Manual.* IPC stands for the Illustrated Parts Catalogue, which is the reference document specific to aircraft type which describes in comprehensive detail every component. "When equipment breaks down or requires routine maintenance, maintenance personnel need to be able to figure out what spare parts are required to complete the work" [34]. Spare parts information is taken from the IPC document;
- *AMM Manual.* The Aircraft Maintenance Manual (AMM) is a document which details the steps of maintenance tasks carried out on an aircraft [35]. Mechanics, regulatory officials, and aircraft and component manufacturers refer to the relevant AMM of the aircraft before they proceed further with the maintenance or repair of the aircraft or its systems;
- *Reference Video.* A tutorial video that contains an illustration of certain procedures on how to perform an action. Videos are taken from real aircraft maintenance sites, demonstrating expert engineers working on a specific instruction;
- *Instructions.* Instructions in text and audio format summarize a subtask and list all that needs to be done in a stage. Audio is played to give a hearing perception, which is convenient when a user is located far from the text panel. At the same time, the instructions panel contains the content of instruction with all referenced objects.

As can be seen in Figure 3, all materials in the subtask content are part of the knowledge base, which is a structured knowledge representation that has all indexes to the required data. A user performing a subtask controls workflow and can go back and forth between stages, where mixed reality content supports and enhances the learning process. 

Figure 1 shows a capture of a trainee that wears a HoloLens 2 and works on the lower side strut removal subtask of the landing gear removal task. The capture in Figure 1 was made using Spectator View [27] which is an augmented reality product that enables viewing HoloLens experiences from other devices (in this case tablet). Thus, the training process and its demonstration are shared among viewers, giving them a chance to have collaborative learning. Multiple trainees using mobile devices such as smartphones, tablets, and smart glasses can simultaneously share the space and view the maintenance lessons demonstrated by a supervisor in real-time. Viewing virtual objects from different angles and perspectives, participants of aircraft metaverse can interact with 3D assets and have hands-on experience. 

**Figure 4:**
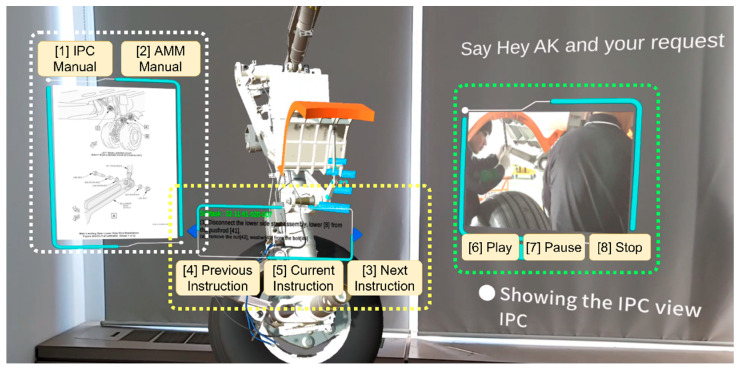
Snapshot of MR maintenance and the speech interaction module reference functions from HoloLens 2 point of view.

To look from a first-person perspective, Figure 4 is a snapshot of the developed MR application in HoloLens 2. As can be seen, the 3D model of the landing gear is illustrated in the center, instructions to perform subtasks are played at the beginning of the stage using smart glasses speakers and shown in the special instruction panel in the center bottom; the IPC or AMM manuals are displayed in the manual panel on the left; the reference video is played in the media player on the right. The virtual objects located in the space are scanned by HoloLens 2; therefore, to analyze, a user may physically come closer towards 3D objects or use gestures to virtually drag 3D assets. Certain parts of the main 3D object have annotations with an identification number and name which are mentioned in the manuals (i.e., for "Remove Nut [42]", the annotation "Nut [42]" is placed onto the 3D virtual object). This makes following instructions and cross-referencing manuals easier for trainees.

Our proposed application for aircraft maintenance education has similar points with other works in this field; however, it delivers considerable improvements and novelties. The authors of [4] have a similar view on aircraft maintenance, having their application developed for use in laptop and mobile phone, but we applied modern HoloLens 2 smart glasses, which is much more effective since there is no need to hold a device. To use manuals, [4] applied AR markers that overlay 3D illustrations on top of the paper; however, we totally eliminate having to work with physical manuals, migrating them into the metaverse. In the case of [8], which presented an AR system for training and assisting in maintaining equipment in the industrial context, the authors presented the work with a head-mounted display with similar approaches; however, special equipment was required, such as a special stand with a camera, belt, and PC for processing. In our case, Microsoft HoloLens 2 replacing all these, creating a powerful independent environment that works in real-time. Overall, comparing related works in the field of MR training, we present new methods such as mixed reality visualizations and legacy manuals into the metaverse at the same time. What is more, we have a unique feature to share the working space of the maintenance process among other users, allowing them to engage in interactive education. In addition, moving forward in effective interaction with virtual objects, we built a custom voice interaction module, which creates opportunities beyond the available technical functions of a device.

To provide users with a smooth way to control the flow, we should take into consideration the nature of maintenance work. Since trainees and field engineers use both hands and need quick access to certain information, speech interaction is an effective way to control the workflow (i.e., to move between subtasks—command "next subtask"). Thus, to develop a speech interaction module, first, the main reference functions to be invoked need to be defined.

### 3.2. Reference Functions and Requirements of Speech Interaction

Overall, the main support for the mixed reality MRO should be in process navigation and content management. We underlined supportive functions and divided them into eight categories. Figure 4 is a snapshot of the MR maintenance application from the user’s point of view, which is deployed in the Microsoft HoloLens 2 smart glasses. As can be seen in Figure 4, using dashed areas, we selected panels that should be managed during operation, and the following functions need to be called with speech commands:

1. *IPC Manual.* The IPC document needs to be displayed in the manuals panel (see Figure 4);
2. *AMM Manual.* The AMM document needs to be displayed in the manuals panel;
3. *Next Instruction.* The required content for the next stage is shown;
4. *Previous Instruction.* The required content for the previous stage is shown;
5. *Current Instruction.* The required content for the current stage is reshown;
6. *Play.* The media player runs a reference video;
7. *Pause.* The media player pauses a reference video;
8. *Stop.* The media player closes a reference video.

Regarding specific characteristics of aircraft maintenance, the process involves multiple professionals, and each person may have their own style of voice interaction. Thus, the speech interaction module should be flexible to capture various speech requests that may refer to the same command. Moreover, users of the application are Korean and English-speaking trainee technicians who deal with aviation-specific manuals written in English, and depending on the person, one might use pure English, Korean, or intermixed-language speech command for interaction. Therefore, all mentioned types of requests should be handled.

Along with taking speech requests, the module should give corresponding responses as well. An engineer should clearly know that a speech request is understood and proceeded, or it needs to be repeated. Thus, the module should give the response message in text and audio formats. To have the best interaction experience, communication between a user and the system should be in the same language; thus, the appropriate feedback (audio and text) by the application should proceed in the language it was requested.

Accordingly, based on the above-mentioned requirements, we proposed a speech interaction module that uses a deep learning model that maps spoken commands to the defined command types and at the same time identifies the language of request to proceed with corresponding feedback. To build and train a deep learning model, which is a data-driven approach, we first collected a dataset.

## 4. Dataset for Speech Interaction Module

**Figure 5:**
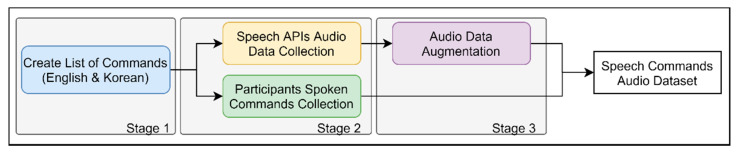
Dataset collection stages.

Dataset creation and collection for our task was performed in three stages (see Figure 5). First, we analyzed the semantic structure of user commands in English and Korean and created a list of potential user requests. Second, based on the list, we collected audio recordings of these speech commands using Text-to-speech (TTS) application programming interface (API) services and asked several people to speak them out. Third, in order to bridge the gap between API and human recordings, we applied various audio augmentation techniques to the TTS-generated data.

### 4.1. Speech Commands

**Figure 6:**
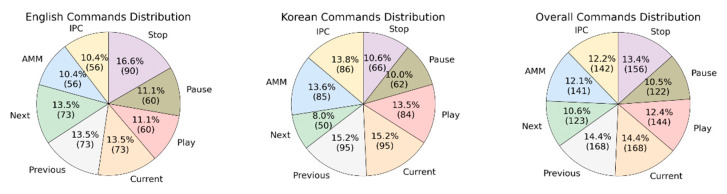
Speech Commands Distribution.

To develop a robust and flexible voice interaction module, it is essential to consider the nature of user requests. Therefore, we analyzed variations in speech commands and semantics for each command type (see Section 3.2). Different users use various combinations to address the same functionality. For example, "next instruction", "display the next instruction", "can you show me the next task" map to the same command—"next instruction". Moreover, end-users of the application use English and Korean languages to have a voice interaction with the MR application. Taking into consideration all these factors and goals, we made a list of potential user speech requests based on command type and language. Figure 6 summarizes the distribution of created speech commands.

Overall, there are 541 commands in English and 623 in the Korean language that mapped to one of eight command types. As can be seen from the charts, the distribution of commands is nearly balanced, having on average about 145 commands for each command type in English and Korean languages in total. In the next stage, we recorded audio files for all created commands.

### 4.2. Audio Data

The audio dataset collection is based on created speech commands. We used two methods to record speech audio: using existing text-to-speech (TTS) API services to create audio recordings, and employing people to record spoken commands. 

For most of the audio collection, we applied API services since this is the cheapest approach. The ones we used were Google Cloud text-to-Speech [36], Microsoft Azure text-to-speech [37], Naver speech synthesis [38], and Kakao speech API [39]. In total, we applied 83 voices—60 English and 23 Korean.

Next, we involved people to record spoken commands: 15 volunteers for English language commands and seven participants for Korean. 

**Figure 7:**
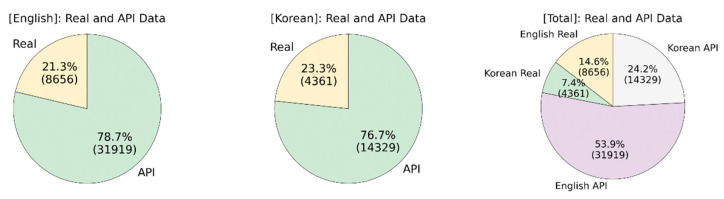
Audio Data Distribution.

Overall, for English commands, we have 60 API, and 15 real people voices, which makes 40,575 (75 × 541) recordings, and for Korean ones, 23 API, and seven real voices, which is 18,690 (30 × 623). Therefore, overall, we collected 59,265 audio recordings as a dataset for all types of commands. Those recordings have a duration of about one to four seconds, which makes the dataset about 24.7 hours long. The voices of API and volunteers have different accents and originated from countries including the USA, Australia, Canada, India, Ireland, United Kingdom, Korea, Uzbekistan. Thus, the dataset has a wide variety of spoken examples for the same phrases, which makes the speech model adapted to variations. Figure 7 summarizes the amount and distribution of audio data.

As can be seen from the charts in Figure 7, API audio data make about 78.1% of the dataset, where the rest 21.9% are spoken commands of employed people. The amount of Korean data is two times less than English, but the proportion of API and real data remains similar. However, having speech API-synthesized audio recordings has its own issues.

### 4.3. Data Augmentation

Text-to-speech API-generated audio recordings differ from the recordings of participants, and we solved this issue using data augmentation. Generally, data augmentation itself is a method to improve the performance of deep neural networks since it helps to generalize a model and avoid overfitting [40], however, we used audio augmentation to bridge the gap between distributions of real and synthesized data. Overall, we found two main issues to address. 

First, API-generated data has no background noise. Usually, when audio is recorded so-called white noise is always present in a recording. However, in the case of synthetic recordings, the audio is too clear, which makes API data distribution vary from real data. To solve this issue, we manually added various types of background noise (i.e., white noise, street noise, crowd noise, etc.) to the API-generated data to make them more realistic. As in [13], we added noise with random volume to have more variations. 

Second, text-to-speech API data has a static playback starting point. In recordings made by humans, the actual point of time when a person begins to speak varies from 0.5 to 1.5 s. This makes people’s audio recordings diverse. In contrast, in the case of API data, the starting point of playback is static and usually starts at zero seconds, which is unrealistic in comparison with human data. To handle this problem, we randomly shifted each API recording to have a diverse beginning time of playback. This approach made API audio recordings sound even more like real ones.

The above-mentioned audio augmentation techniques made the real and API distributions closer, and recordings of both types sound similar now. Hence, by finishing the dataset collection stages described in Figure 5, as a result, we have a speech commands dataset in the form of audio files, each having a sampling rate of 16 kHz and a bit rate of 256 kbit/s. Next, we created and trained our speech model.

## 5. Speech Interaction Module

The speech interaction module in our mixed reality maintenance education plays the role of an assistant that can help to navigate during the maintenance process and deliver and illustrate the required materials just at a speech request, which provides the ability for multi-tasking, and a feeling of supervision. Figure 2 shows the speech interaction flow. A user makes a speech request and the speech interaction module identifies the command and language it was spoken in. Next, based on the predictions, MR provides requested materials and takes a trainee further in stages by giving corresponding feedback based on the requested command and language.

**Figure 8:**
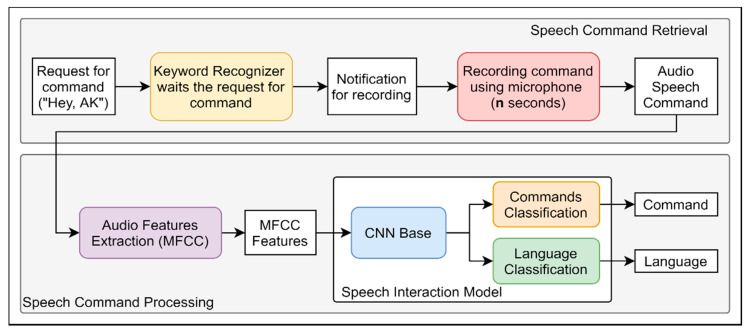
Speech interaction module inference flow.

The proposed speech interaction module action flow consists of two steps: speech command retrieval and speech command processing (see Figure 8). In the speech command retrieval, the module first accepts a request for a command. Following approaches of voice assistants such as Apple Siri [41] ("Hey, Siri") or Google [42] ("Hey, Google"), we used our own trigger "Hey, AK" (AK is short for Augmented Knowledge Corp., the company that supported our work and provided Microsoft HoloLens 2 smart glasses and virtual 3D assets of the Boeing 737). Thus, whenever a user wants to give a command, "Hey, AK" should be spoken, and it is then detected by the keyword recognizer, which gives the notification sound that denotes recording, and next triggers the audio recording using a microphone with a duration of n seconds. In our case, four seconds was sufficient for an average request. The speech command retrieval produces an audio speech command that is processed in the next stage. 

In the speech command processing step, we extract audio features. We use mel frequency cepstral coefficients (MFCCs) as audio representation. Next, using our speech interaction model, we predict the requested command type and the language in which the command was spoken. Our deep learning network is divided into three parts: CNN base (convolutional neural network architecture for audio features learning), commands, and language classification (classifier networks that make predictions according to audio features learned in CNN base). Based on the command classification result, the MR application performs a particular action, and voice feedback given in the language predicted by the language classifier (i.e., if a user makes a speech request in Korean, the response from the application will be in Korean, accordingly). In the case of when a request mixes a few Korean words and technical English terms or any Korean word is pronounced, the model will classify the language as Korean. 

To train the model on our custom dataset, which consists of complex speech requests in two languages that sound completely different, we extracted audio features as data representation and presented training methods that played a significant role in network convergence. Let us start with audio data representation.

### 5.1. Audio Features Extraction

There are many ways to extract audio features; however, based on our research, MFFCs or mel frequency cepstral coefficients tend to suit the best for linguistic content analysis [26]. The job of MFCCs is to represent a sound’s power spectrum produced by a human’s vocal tract. MFCCs preserve information about spoken content and discards carried background noise. MFCC features are robust and reliable to a speaker’s variations and recording conditions [43]. What is more, MFCCs correlate audio features well, which perfectly suits the deep learning approach [13]. Therefore, MFCCs are widely used in recent speech recognition methods. Thus, taking advantage of mel frequency cepstral coefficients, we extracted audio features from spoken commands.

**Figure 9:**
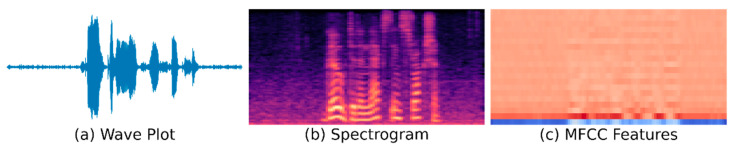
Audio features illustration: (**a**) is wave plot of an audio signal, (**b**) is the corresponding Spectrogram visualization of the audio, and (**c**) is a plot of MFCC features extracted from the audio.

Figure 9 shows the illustration of audio and its feature forms. Taking the example of the speech command "Go to the next instruction", Figure 9a is a wave plot of the recorded audio, Figure 9b is its representation in spectrogram form, and finally, Figure 9c is the MFCC features of audio we applied in our work. 

To be precise, in our work, we applied the following parameters that have been used to extract MFCCs from audio:

- Number of MFCC features: 13;
- Length of the fast Fourier transform (FFT) window: 512;
- Hop length: 512;
- Number of mel bands to generate: 40.

Each audio file that we processed was scaled down to 204x13 dimensions, where 13 is the number of MFCCs, and 204 is the resultant number of frames after sampling with the hop length. Basically, 12 to 20 cepstral coefficients are typically optimal for speech analysis and an increasing number of MFCCs give more details and, at the same time, results in more complexity in the models [44]. Empirically, the mentioned parameters worked best in our task. Moving on, according to the audio features dimensions, we built the deep learning model for the speech interaction module.

### 5.2. Model Architecture

**Figure 10:**
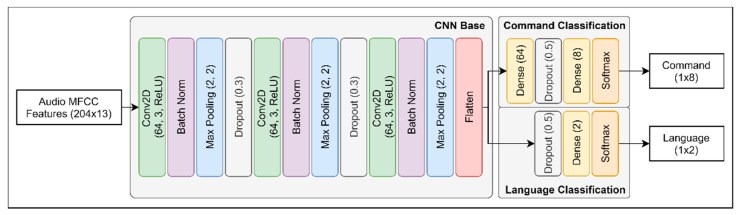
Speech interaction module network architecture.

The architecture of the proposed speech interaction module network consists of three parts (see Figure 10). First is the convolutional neural network (CNN) base, which takes the audio features vector of MFCCs passed as an input. Since we deal with two-dimensional MFCC features, we applied respective 2D dimensions for the convolutional layer filters. The CNN base consists of stacks of convolutional, batch normalization, max pooling, and dropout layers. As can be seen in Figure 10, Conv2D has three parameters, which are the number of filters (64), kernel size (3), and the activation function applied (ReLU) at the end of the layer. In the case of max pooling, parameters denote pool size and stride, which are 2 for both. In addition, the dropout shows the dropout rate equals 0.3. At last, the flatten layer reshapes the input to be one-dimensional to pass the output for classification. Overall, the CNN base gathers features present in the audio, and passes them for classification parts. 

The second part is the command classification. This part of the network is devoted to the classification of any command requested by a user. The stack of layers consists of the fully connected layers, which are denoted by dense and the number of units in it (64 or 8), the dropout (0.5), and the Softmax activation function, which gives the final output vector of shape 1 × 8, where 8 is a representation for each command type.

Third, the language classification layers, that take the output from the CNN base to predict the language in the input command was spoken. It is similar to the commands classification part; however, it has a lower number of dense layers. The output vector of this part is the predicted language with the shape of 1x2, where 2 is the score for each language (English and Korean). 

All three parts combined make the architecture of the proposed model, which consists of only 181,066 trainable and 384 non-trainable parameters, respectively. However, building the architecture is only one part of the work; the next thing is to find proper training techniques to make it work with complex characteristics of the dataset.

### 5.3. Model Training

Training deep learning models requires a proper pick of methods based on the dataset and overall task. In our work, we use data in English and Korean languages with diverse spoken commands. In addition, we want the model to work in a real-world environment, which has various background noises and new human voices with their unique characteristics. Thus, the most essential goal we have is to avoid overfitting. To handle this task, we applied weight decay, which applies regularization for model weights [45]. This approach improves the generalization of the model and makes it perform well on data that is not present in the dataset.

Next, we found that using the cosine annealing scheduler [46] for setting a proper learning rate helped a lot for the model to converge [14,47]. The general working principle of the cosine annealing scheduler is setting the learning rate starting with a large value that is relatively rapidly decreased to a minimum value before being increased rapidly again [46]. With each restart, the scheduler tries to start with the weights that contribute to the training most; therefore, this technique is referred to as a "warm restart".

Talking about parameters such as loss and optimizer, for each command and language classification part, we applied categorical cross entropy loss, having loss weights to be equally distributed. Moreover, Adam optimizer was applied, having the following hyperparameters: Beta 1—0.9, Beta 2—0.999, Epsilon—1e-07. 

Overall, the mentioned techniques helped the model to converge and learn diverse features of the dataset. In the next section, we discuss the hardware and software setup, and the time taken for training and inference of the network.

### 5.4. Hardware and Software Environment

To set up a deep learning development environment for our work, we used a PC with the following specifications and software:

- OS: Ubuntu 18.04.4 LTS 64-bit;
- CPU: Intel® Core™ i3-8100 CPU @ 3.60GHz × 4;
- RAM: 8 GB;
- GPU: GeForce RTX 2080 Ti/PCIe/SSE2 11 GB;
- Framework: Tensorflow 1.13.2 & Keras 2.2.4-tf [48].

The proposed network was trained for 50 epochs, and with all the cross-validation and checkpoint model savings, training time took about six minutes, reaching a training accuracy of 99% by the end of the process.

The created model in the h5 extension was exported to ONNX format and imported to the Unity framework [49] with Barracuda 1.12.1 (cross-platform neural network inference library for Unity [50]), where the mixed reality application is built. After exporting to the corresponding format, the model has a 716KB disk memory allocation size. For the development of the mixed reality application that later deployed to HoloLens 2, we used a PC with the following specifications and software:

- OS: Windows 10 Pro 64-bit 20H2 19042.804;
- CPU: Intel® Core™ i5-9400F CPU @ 2.90GHz × 6;
- RAM: 16GB;
- GPU: GeForce RTX 2080 Ti/PCIe/SSE2 11GB;
- Framework: Unity 2019.3.15f & Barracuda 1.12.1.

Next, the application with the speech interaction module deployed to Microsoft HoloLens 2 (untethered holographic computer or new generation smart glasses [10]) to check the inference time of the model, which was about 40 to 60 milliseconds to process a speech request. Overall, the presented details show that the proposed model requires a small disk size and has a real-time inference. Next, we performed the evaluation process to assess the performance of the model on test data.

## 6. Evaluation of Speech Interaction Module

Once the model is trained, it needs to be assessed based on certain evaluation metrics regarding different characteristics of machine learning algorithms [51]. Since our network deals with classification task, accordingly, we used classification metrics such as accuracy, precision, recall, and F1-Score. We described in detail the intuition behind these assessment methods and how they work and summarized the results of experiments. The evaluation process started with the testing data preparation.

### 6.1. Test Data

To perform the evaluation process of the proposed speech interaction model, first, we created the test sets. We randomly picked 2000 audio samples from our dataset. The network was not trained on these audios; however, it might see the other samples of the same voice. We denoted this set as test data. Next, since we have two languages, we decided to assess performance for each one with real people’s data, which the network has not seen. Therefore, we applied two sets of spoken commands by humans for English and Korean data. One set consists of all spoken commands by a person. Thus, for English data, we used 541 test audio samples, and 623 for Korean. We denoted these sets as test English speaker and test Korean speaker. Next, we discuss the evaluation metrics that were be used for testing.

### 6.2. Evaluation Metrics

In this work, we used accuracy, precision, recall, and F1-Score classification metrics to evaluate the speech recognition model. First, let us define notations:

- *True Negative (TN)*—correct prediction: The actual value was false, and the model predicted false;
- *False Positive (FP)*—prediction error: The actual value was false, and the model predicted true;
- *False Negative (FN)*—prediction error: The actual value was true, and the model predicted false;
- *True Positive (TP)*—correct prediction: The actual value was true, and the model predicted true.

Based on these notations, we explained the evaluation metrics below:

- **Accuracy**: the ratio of accurately classified predictions to the total number of predictions (see Formula (1)).
  Accuracy = (TP + TN)/(TP + TN + FP + FN)(1)
  Accuracy is a technique to evaluate the classification model and works best in the case of a balanced dataset [52]. Since our test data followed the overall distribution of speech commands (see Figure 6), which is nearly balanced, it is appropriate to use accuracy.
- **Precision**: out of the predictions that are positive, how many of them are really positive. Generally, it shows often the model makes correct predictions. In Formula (2), we can see that precision is equal to number of true positives divided by the sum of true positives and false positives.
  Precision = TP/(TP + FP)(2)
  Thus, if the precision is low, then the model makes false positives a lot.
- **Recall**: out of all test data that belong to a particular class (i.e., class A), how many predictions the model predicted as class A. Basically, when the true class is A, how often the model predicts correctly. In Formula (3), recall equals the number of true positives divided by the sum of true positives and false negatives.
  Recall = TP/(TP + FN)(3)
  When recall is low, then the number of false negatives is high.
- **F1-score**: This is a combination of precision and recall scores, making a weighted average between them. F1-score is described in Formula (4):
  F1-score = 2 × (Precision × Recall)/(Precision + Recall)(4)
  F1-score is useful when having an unbalanced test dataset. A high F1 score means that you have low false positives and low false negatives, thus, correctly identifying true classes.
- **Confusion Matrix**: a table that shows a visualization of the performance of an algorithm. Each row of the matrix represents the samples of a predicted class, whereas each column shows the instances in an actual class (or vice versa) [53]. To easily see the good performance of the matrix, it should be diagonal, so that true and predicted classes must match, and the rest of positions are zero.

These classification metrics were used to evaluate the model. We described experimental results in the next section.

### 6.3. Results

Based on the performance of the proposed model experimented on test data, we evaluated our network using classification metrics such as accuracy, precision, recall, and F1-score. Since we have two classification outputs (command and language), we evaluated each separately.

Table 1 summarizes the assessment for the commands classification part.

As can be seen, performance on test data is near perfect with a 99% success rate in each metric. However, in the case of English and Korean test voices, results are about five percent lower but still great with 94.5% on average for each metric. Results with test data show better performance because the network might have seen some voice characteristics of test samples (other data of the same voice); however, still, it was not trained on this data. In the case of test English and Korean data, these voice features were totally new, and the network has not seen similar audio samples of these voices. To sum up this experiment, the results of the best model in the form of the confusion matrix are illustrated in Table 2 for test data, Table 3 for test English data, and Table 4 for test Korean data, which shows the exact amount of correct and incorrect predictions.

Next, we demonstrated the results of the language classification part in Table 5.

This experiment shows that, for all test sets, the proposed speech commands classification network performed nearly perfectly, with about 99% for all metrics. Since we have only two languages, this task is a binary classification; therefore, it was easily handled by the network.

The experiment results demonstrated, that for our task, we collected a proper dataset that was enough to train the proposed network and satisfy our needs. In addition, the model successfully passed the evaluation and tended to identify the commands’ type accurately. In all presented metrics (accuracy, precision, recall, and F1-Score), the model performed nearly similar, which means the network can successfully predict voice commands regarding any class.

## 7. Conclusions and Future Works

To conclude, in this work, we performed a forward step towards the aircraft maintenance metaverse, creating a mixed reality digital place for Boeing 737 maintenance education enhanced with a speech interaction module in the Microsoft HoloLens 2 smart glasses. Using a virtual 3D twin of the aircraft and supportive aviation-specific materials, the proposed MR application provides guidance and supervision during the maintenance process, delivering trainees step-by-step instructions, mixed reality visualizations, reference videos, and aircraft operation manuals to complete MRO tasks. Moreover, the developed speech interaction module, which is based on the deep learning model integrated into the HoloLens 2, enables users to intuitively deal with virtual objects in the aircraft metaverse and seamlessly navigate the maintenance process using speech commands without being distracted from the workflow by pressing buttons or using hand gestures, which is essential when hands are busy with equipment.

To develop the speech interaction model, firstly, based on the defined requirements of the application, we collected a bilingual speech commands dataset in English and Korean languages with domain-specific vocabulary, which included a variety of requests to invoke functions. Secondly, for a core of the speech interaction module, we built and trained a unique deep learning-based CNN network with two outputs that recognizes a referenced command and its language from extracted audio features of the user’s speech request. Having a small number of training parameters and low memory size, the model is lightweight and inferences in real-time, which perfectly suits standalone devices such as smart glasses. It was successfully integrated into the Microsoft HoloLens 2, replacing built-in voice interaction. Lastly, to assess the performance of the speech interaction module, we conducted the evaluation using classification metrics and various test data sets. The experimental results based on English and Korean test data showed that the speech interaction model can accurately recognize commands along with the language, achieving on average 95.7 and 99.6% on the F1-Score metric for command and language prediction, respectively. As a result, the proposed speech interaction module in the aircraft maintenance metaverse further improved the aircraft maintenance training by giving intuitive and efficient control over the operation, empowering voice interaction in mixed reality smart glasses.

In the future, we want to further move forward in developing a metaverse for aircraft maintenance and education, by allowing engineers and trainees located in different places to simultaneously engage with MR experiences in created virtual spaces. What is more, we plan to enlarge the variety of referencing speech commands and application functions to deal with complex interactions based on contextual information present in a certain scene, where questions and answers are given according to the knowledge base.

## Figures and Tables

**Table 1:** Commands Classification Results.

Data	Accuracy	Precision	Recall	F1-Score
Test Data	0.99	0.99	0.99	0.99
Test English Speaker	0.94	0.96	0.939	0.941
Test Korean Speaker	0.95	0.944	0.942	0.942

**Table 2:** Confusion Matrix for Speech Commands Classification Results of Test Data.

		Predicted Results							
		IPC	AMM	Next	Previous	Current	Play	Pause	Stop
True Data	IPC	216	0	0	0	0	0	0	0
	AMM	0	225	1	0	0	1	1	0
	Next	0	0	250	0	0	0	0	0
	Previous	0	0	0	253	1	0	0	0
	Current	0	0	1	0	295	0	0	0
	Play	0	0	0	0	0	247	0	0
	Pause	0	1	0	0	0	0	210	6
	Stop	0	0	0	0	1	5	1	285

**Table 3:** Confusion Matrix for Speech Commands Classification Results of Test English Data.

		Predicted Results							
		IPC	AMM	Next	Previous	Current	Play	Pause	Stop
True Data	IPC	56	0	0	0	0	0	0	0
	AMM	0	56	0	0	0	0	0	0
	Next	0	0	73	0	0	0	0	0
	Previous	0	0	0	73	0	0	0	0
	Current	0	0	0	0	73	0	0	0
	Play	0	0	0	0	0	60	0	0
	Pause	0	1	0	0	0	1	33	26
	Stop	0	0	0	0	0	1	2	87

**Table 4:** Confusion Matrix for Speech Commands Classification Results of Test Korean Data.

		Predicted Results							
		IPC	AMM	Next	Previous	Current	Play	Pause	Stop
True Data	IPC	86	0	0	0	0	0	0	0
	AMM	0	85	0	0	0	0	0	0
	Next	0	0	50	0	0	0	0	0
	Previous	0	0	0	93	1	0	1	0
	Current	0	0	0	2	92	1	0	0
	Play	0	0	2	1	1	77	1	2
	Pause	1	0	0	1	1	1	55	3
	Stop	0	0	0	1	5	3	5	52

**Table 5:** Language Classification Results.

Data	Accuracy	Precision	Recall	F1-Score
Test Data	0.998	0.998	0.998	0.998
Test English Speaker	0.98	1.00	0.98	0.99
Test Korean Speaker	0.99	1.00	0.99	1.00

## Data Availability

Data available on request due to privacy restrictions.

## References

[1] Baszucki D. The Metaverse Is Coming. https://www.wired.co.uk/article/metaverse.

[2] Masters N.B., Shih S.-F., Bukoff A., Akel K.B., Kobayashi L.C., Miller A.L., Harapan H., Lu Y., Wagner A.L. (2020). Social Distancing in Response to the Novel Coronavirus (COVID-19) in the United States. PLoS ONE.

[3] How Much Do Boeing Aircraft Cost?. https://simpleflying.com/how-much-do-boeing-aircraft-cost/.

[4] Christian J., Krieger H., Holzinger A., Behringer R., Stephanidis C. (2007). Virtual and Mixed Reality Interfaces for E-Training: Examples of Applications in Light Aircraft Maintenance. Proceedings of the Universal Access in Human-Computer Interaction. Applications and Services.

[5] Eschen H., Kötter T., Rodeck R., Harnisch M., Schüppstuhl T. (2018). Augmented and Virtual Reality for Inspection and Maintenance Processes in the Aviation Industry. Procedia Manuf..

[6] Fonnet A., Alves N., Sousa N., Guevara M., Magalhães L. Heritage BIM Integration with Mixed Reality for Building Preventive Maintenance. Proceedings of the 2017 24o Encontro Português de Computação Gráfica e Interação (EPCGI).

[7] Espíndola D.B., Pereira C.E., Henriques R.V.B., Botelho S.S. (2010). Using Mixed Reality in the Visualization of Maintenance Processes. IFAC Proc. Vol..

[8] Schwald B., Laval B., Sa T., Guynemer R. (2003). An Augmented Reality System for Training and Assistance to Maintenance in the Industrial Context. J. WSCG.

[9] Silva H., Resende R., Breternitz M. Mixed Reality Application to Support Infrastructure Maintenance. Proceedings of the 2018 International Young Engineers Forum (YEF-ECE).

[10] HoloLens 2—Overview, Features, and Specs|Microsoft HoloLens. https://www.microsoft.com/en-us/hololens/hardware.

[11] Voice Input—Mixed Reality. https://docs.microsoft.com/en-us/windows/mixed-reality/design/voice-input.

[12] LeCun Y., Bengio Y., Hinton G. (2015). Deep Learning. Nature.

[13] Solovyev R.A., Vakhrushev M., Radionov A., Romanova I.I., Amerikanov A.A., Aliev V., Shvets A.A. Deep Learning Approaches for Understanding Simple Speech Commands. Proceedings of the 2020 IEEE 40th International Conference on Electronics and Nanotechnology (ELNANO).

[14] Jansson P. (2018). Single-Word Speech Recognition with Convolutional Neural Networks on Raw Waveforms. Ph.D. Thesis.

[15] Sharmin R., Rahut S.K., Huq M.R. (2020). Bengali Spoken Digit Classification: A Deep Learning Approach Using Convolutional Neural Network. Procedia Comput. Sci..

[16] Gu Y., Li X., Chen S., Zhang J., Marsic I. (2017). Speech Intention Classification with Multimodal Deep Learning. Adv. Artif. Intell..

[17] Hickey C., Zhang B.T. (2020). Classifying Verbal Commands Using Convolutional Neural Networks and Mel-Spectrographic Sound Representations. J. Korean Inf. Sci. Soc..

[18] Ajmera P., Sinha H., Awasthi V. (2020). Audio Classification Using Braided Convolutional Neural Networks. IET Signal Process..

[19] Mamyrbayev O., Mekebayev N., Turdalyuly M., Oshanova N., Medeni T.I., Yessentay A., Mekebayev N. (2020). Voice Identification Using Classification Algorithms. Intelligent System and Computing.

[20] Nguyen Q.H., Cao T.-D. (2020). A Novel Method for Recognizing Vietnamese Voice Commands on Smartphones with Support Vector Machine and Convolutional Neural Networks. Wirel. Commun. Mob. Comput..

[21] Warden P. (2018). Speech Commands: A Dataset for Limited-Vocabulary Speech Recognition. arXiv.

[22] Kim Y. (2014). Convolutional Neural Networks for Sentence Classification. Proceedings of the 2014 Conference on Empirical Methods in Natural Language Processing (EMNLP).

[23] Lu H., Zhang H., Nayak A. (2020). A Deep Neural Network for Audio Classification with a Classifier Attention Mechanism. arXiv.

[24] Kamatchy B., Dhanalakshmi P. (2020). A Deep Learning CNN Model for TV Broadcast Audio Classification. Int. J. Eng. Res. Technol..

[25] Nanni L., Costa Y.M.G., Aguiar R.L., Mangolin R.B., Brahnam S., Silla C.N. (2020). Ensemble of Convolutional Neural Networks to Improve Animal Audio Classification. EURASIP J. Audio Speech Music Process..

[26] Mel Frequency Cepstral Coefficient (MFCC) Tutorial. http://practicalcryptography.com/miscellaneous/machine-learning/guide-mel-frequency-cepstral-coefficients-mfccs/.

[27] Spectator View|MixedReality-SpectatorView Documentation. https://microsoft.github.io/MixedReality-SpectatorView/README.html.

[28] Müller J., Rädle R., Reiterer H. (2016). Virtual Objects as Spatial Cues in Collaborative Mixed Reality Environments: How They Shape Communication Behavior and User Task Load. Proceedings of the 2016 CHI Conference on Human Factors in Computing Systems.

[29] Boeing 737. https://en.wikipedia.org/w/index.php?title=Boeing_737&oldid=1006373038.

[30] Plumley C.S.J.M. Aircraft Operating Series—Aircraft Operating Expenses. https://www.opshots.net/2015/04/aircraft-operating-series-aircraft-operating-expenses/.

[31] Gogate M., Dashtipour K., Hussain A. Visual Speech In Real Noisy Environments (VISION): A Novel Benchmark Dataset and Deep Learning-Based Baseline System. Proceedings of the INTERSPEECH.

[32] Salamon J., Jacoby C., Bello J.P. A Dataset and Taxonomy for Urban Sound Research. Proceedings of the 22nd ACM International Conference on Multimedia (ACM-MM’14).

[33] Martinez J., Perez H., Escamilla E., Suzuki M.M. Speaker Recognition Using Mel Frequency Cepstral Coefficients (MFCC) and Vector Quantization (VQ) Techniques. Proceedings of the CONIELECOMP 2012, 22nd International Conference on Electrical Communications and Computers.

[34] Make Maintenance More Visual Using Illustrated Part Catalogue. https://www.intellinetsystem.com/blog/more-visual-using-illustrated-part-catalogue.htm.

[35] Intech|AMM/TSM. http://www.intech.eu/en/technical-publications/offer/amm-tsm.

[36] Text-to-Speech: Lifelike Speech Synthesis. https://cloud.google.com/text-to-speech.

[37] Text to Speech|Microsoft Azure. https://azure.microsoft.com/en-us/services/cognitive-services/text-to-speech/.

[38] Naver Cloud Platform (Text-To-Speech)—API Reference. https://apidocs.ncloud.com/en/ai-naver/clova_speech_synthesis/tts/.

[39] Speech API|Kakao API. https://speech-api.kakao.com/.

[40] Wang J., Kim S., Lee Y. Speech Augmentation Using Wavenet in Speech Recognition. Proceedings of the ICASSP 2019—2019 IEEE International Conference on Acoustics, Speech and Signal Processing (ICASSP).

[41] Siri. https://www.apple.com/siri/.

[42] Google Assistant, Your Own Personal Google. https://assistant.google.com/.

[43] Dave N. (2013). Feature Extraction Methods LPC, PLP and MFCC in Speech Recognition. Int. J. Adv. Res. Eng. Technol..

[44] Poorjam A.H. Why We Take Only 12-13 MFCC Coefficients in Feature Extraction?. https://www.researchgate.net/post/Why_we_take_only_12-13_MFCC_coefficients_in_feature_extraction.

[45] Krogh A., Hertz J., Moody J., Hanson S., Lippmann R.P. (1992). A Simple Weight Decay Can Improve Generalization. Proceedings of the Advances in Neural Information Processing Systems.

[46] Loshchilov I., Hutter F. (2016). SGDR: Stochastic Gradient Descent with Restarts. CoRR.

[47] Jordan J. Setting the Learning Rate of Your Neural Network. https://www.jeremyjordan.me/nn-learning-rate/.

[48] Module: Tf.Keras|TensorFlow Core v2.4.1. https://www.tensorflow.org/api_docs/python/tf/keras.

[49] Technologies U. Unity Real-Time Development Platform|3D, 2D VR & AR Engine. https://unity.com/.

[50] Technologies U. Barracuda. https://docs.unity3d.com/Manual/com.unity.barracuda.html.

[51] Sokolova M., Japkowicz N., Szpakowicz S. (2006). Beyond Accuracy, F-Score and ROC: A Family of Discriminant Measures for Performance Evaluation. AI 2006 Adv. Artif. Intell..

[52] Vakili M., Ghamsari M., Rezaei M. (2020). Performance Analysis and Comparison of Machine and Deep Learning Algorithms for IoT Data Classification.

[53] Confusion Matrix. https://en.wikipedia.org/w/index.php?title=Confusion_matrix&oldid=999451492.

